# Climatic and Soil Factors Shape the Demographical History and Genetic Diversity of a Deciduous Oak (*Quercus liaotungensis*) in Northern China

**DOI:** 10.3389/fpls.2018.01534

**Published:** 2018-10-25

**Authors:** Jia Yang, Lucía Vázquez, Li Feng, Zhanlin Liu, Guifang Zhao

**Affiliations:** ^1^Key Laboratory of Resource Biology and Biotechnology in Western China, Ministry of Education, College of Life Sciences, Northwest University, Xi’an, China; ^2^Biology Department, University of Illinois at Springfield, Springfield, IL, United States; ^3^School of Pharmacy, Xi’an Jiaotong University, Xi’an, China

**Keywords:** demographic history, genetic variation, environmental changes, glacial refugia, temperate tree, *Quercus liaotungensis*

## Abstract

Past and current climatic changes have affected the demography, patterns of genetic diversity, and genetic structure of extant species. The study of these processes provides valuable information to forecast evolutionary changes and to identify conservation priorities. Here, we sequenced two functional nuclear genes and four chloroplast DNA regions for 105 samples from 21 populations of *Quercus liaotungensis* across its distribution range. Coalescent-based Bayesian analysis, approximate Bayesian computation (ABC), and ecological niche modeling (ENM) were integrated to investigate the genetic patterns and demographical history of this species. Association estimates including Mantel tests and multiple linear regressions were used to infer the effects of geographical and ecological factors on temporal genetic variation and diversity of this oak species. Based on multiple loci, *Q. liaotungensis* populations clustered into two phylogenetic groups; this grouping pattern could be the result of adaptation to habitats with different temperature and precipitation seasonality conditions. Demographical reconstructions and ENMs suggest an expansion decline trend of this species during the Quaternary climatic oscillations. Association analyses based on nuclear data indicated that intraspecific genetic differentiation of *Q. liaotungensis* was clearly correlated with ecological distance; specifically, the genetic diversity of this species was significantly correlated with temperature seasonality and soil pH, but negatively correlated with precipitation. Our study highlights the impact of Pleistocene climate oscillations on the demographic history of a tree species in Northern China, and suggests that climatic and soil conditions are the major factors shaping the genetic diversity and population structure of *Q. liaotungensis*.

## Introduction

Past environmental changes, such as orographic events and climatic oscillations during the Quaternary, have left complex imprints on the structure and genetic diversity of present day species (e.g., Petit et al., 2002; Chen et al., 2008; Bai et al., 2010; Zeng et al., 2015; Bagnoli et al., 2016). Additional factors that impact genetic variation are life history traits and effective population sizes (Ortego et al., 2015; Sun et al., 2015; Bai et al., 2018). The genetic diversity and structure of organisms with long generation times, large effective population sizes, and limited dispersal to new environments (e.g., changes caused by glacial-interglacial climatic fluctuations during the Quaternary) tend to show time lags (Gugger et al., 2013; Ortego et al., 2015). By contrast, species with high dispersal potential respond faster to new environments and have the ability to reach early equilibrium (Landguth et al., 2010; Kremer et al., 2012).

Insights from phylogeography and population genetics suggest that stable regions during the glacial period act as species shelters (e.g., refugia) and play an important role in post-glaciation recolonization because some glacial populations may harbor high levels of genetic diversity (Bennett and Provan, 2008; Stewart et al., 2010); in these cases, refugia are ideal habitats to conserve plant genetic diversity and generate unique alleles (Hewitt, 2000; Gugger et al., 2013). During the interglacial period, genetic diversity of any given species could be shaped by population expansion and/or migration to new habitats (e.g., a diffusion toward the high latitude) (Petit et al., 2002; Hewitt, 2004) resulting in different genetic structure scenarios. In one scenario, genetic differentiation between locally adapted and newly established populations, due to a reduction of intraspecific gene flow, would be observed; this pattern would follow a center-periphery model and the relationship between genetic differentiation and geographical distance would show a monotonic increase. Moreover, an isolation-by-distance (IBD) pattern would be expected if populations are in migration-drift equilibrium (Ribera et al., 2011; Ortego et al., 2012). In a second scenario, the spatial genetic structure among populations would be erased via homogenization due to high levels of gene flow between locally adapted and newly established populations, provided that species maintained stable population sizes or expanded gradually (Bai et al., 2010). In a third scenario, an increase in local genetic diversity would be expected if genetic admixture from immigrants of genetically differentiated populations took place (He et al., 2013; Ortego et al., 2015). In a last scenario, increased population differentiation and reproductive isolation would be the result of temporal barriers that limit gene flow; this scenario falls under the “asynchrony of seasons hypothesis” where heterogeneous microenvironments or variation in climate seasonality represent the barriers that cause reproduction isolation (Martin et al., 2009; Quintero et al., 2014). In this situation, local adaptation among conspecific populations could happen due to asynchronous phenologies, and an isolation-by-ecology (IBE) pattern would be expected to account for contemporary patterns of intraspecific genetic differentiation and population structure (Elias et al., 2012; Orsini et al., 2013).

In Northern China, paleogeological evidence suggests the absence of extensive ice sheets during the Quaternary glaciation; however, very cold and dry conditions with a strong winter monsoon occurred during the glacial period and had a remarkable impact on the demographic history and genetic variation of temperate tree species (Yu et al., 2000; Liu et al., 2012). Palaeovegetation reconstructions and limited phylogeographical studies have documented in detail the sites of glacial refugia and the response patterns of temperate trees distributed in Northern China during Quaternary climatic changes (Qian and Ricklefs, 2000; Harrison et al., 2001; Chen et al., 2008; Bai et al., 2010; Zeng et al., 2015; Yang et al., 2016). However, few studies have focused on the effect of past environmental changes on species demography (but see Bai et al., 2018 who estimated the population dynamics of several walnut species based on genomic data including a species distributed in Northern China).

Northern China is a mega-diverse area characterized by complex topography and relatively mild Pleistocene climates. The Yanshan, Taihang, and Qinling mountains form a north–south vegetation transect with many microclimates (Bai et al., 2010); thus, this region offers an excellent opportunity to study the role of environmental changes on species demography and on temporal genetic structure and diversity.

*Quercus liaotungensis* Koidz. (*Quercus* L.) is an important economic and dominant deciduous tree species in Northern China (Du et al., 2007; Wang and Zhang, 2011; Li et al., 2012). Populations of this species are mainly distributed in mountainous areas at the north of the Qinling Mountains, the Liupan Mountain, the Taihang Mountains, and the Changbai Mountains at elevations of *ca.* 1000-2200 m; however, current populations of this oak species are largely fragmented due to anthropogenic interference. Previous studies based on genetic data suggest that *Q. liaotungensis* and its close relative *Q. mongolica* diverged recently, probably at the Pliocene/Pleistocene boundary (Yang et al., 2016). Also, several studies propose that genetic introgression took place between these species; however, distinct gene pools and clear species boundaries between the two oaks have been found (Zeng et al., 2010, 2011; Li et al., 2012; Yang et al., 2016). Prior phylogeographical studies indicate that *Q. liaotungensis* migrated to the Qinling Mountains and adjacent areas during the LGM (Zeng et al., 2011; Yang et al., 2016); during this period, these mountains were considered suitable plant refugia due to their heterogeneous topography and climate (Ying, 1994). Consequently, climate changes during the glacial-interglacial period have affected the distribution of *Q. liaotungensis*; this situation provides an excellent opportunity to understand the effects of extrinsic factors on the population structure and genetic diversity of this species, as well as the demographic response of temperate tree species to environmental changes in Northern China.

In this study, we integrated genetic information from chloroplast and nuclear sequences, coalescent-based simulations, and ecological niche modeling (ENM) to reconstruct demography patterns of *Q. liaotungensis*. We also estimated the association between effective population size changes and past climatic oscillations. Additionally, we used correlation analyses to seek potential relationships between temporal genetic structure and genetic diversity, and geographical and ecological factors (under the assumption of recent speciation, Yang et al., 2016). We also tested the relative importance of habitat status on species genetic turnover and population differentiation.

## Materials and Methods

### Sample Information and Sequencing of Multiple Loci

**FIGURE 1:**
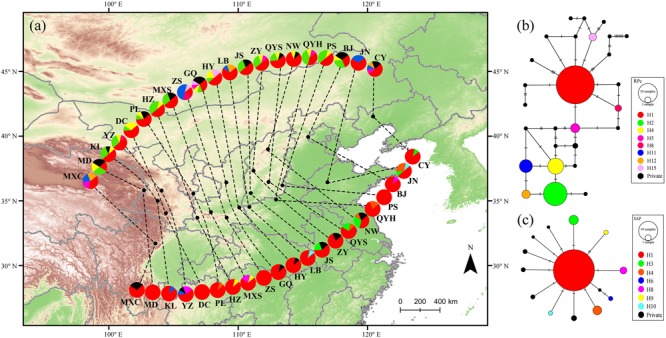
Haplotype distributions **(a)** and networks **(b,c)** of the RPc (above) and SAP (below) genes for *Quercus liaotungensis* in Northern China. Black dots in **(a)** indicate locations of the 21 collected populations of *Q. liaotungensis*. Pie charts are proportional to the numbers of samples in each population (five individuals per site). Colors of pie charts correspond to different haplotypes in the network. Area of the pie in the network is proportional to the number of haplotypes identified.

To ensure representation of genetic information and diverse habitats, we collected 105 individuals from 21 populations throughout the geographical distribution of *Q. liaotungensis* in Northern China (Figure 1 and Supplementary Table S1). All samples from each population were spaced at least 100 m apart, and fresh leaves were collected for DNA extraction. Total genomic DNA was extracted using the Plant Genomic DNA Kit from TIANGEN (TIANGEN, Beijing, China).

We sequenced two single-copy nuclear genes: the CC-NBS-LRR gene involved in resistance to *Phytophthora cinnamomi* (RPc) and a predicted stress-associated protein gene (SAP). These two nuclear genes were selected based on a comparative transcriptome work in three Chinese oak species (Sun and Yang et al., unpublished results). Sequences of four chloroplast regions (*psb*A-*trn*H, *trn*L-*trn*F, *ycf*1, and *mat*K) for the same samples were compiled from our previous research (Yang et al., 2016). PCR protocols were the same as in Yang et al. (2016) and primers used are listed in Supplementary Table S2. All sequences were checked and aligned in BioEdit 7.0.9.0 (Hall, 1999). Potential indels found in the chloroplast regions were treated as informative with exception of the approximate Bayesian computation (ABC) analysis where gaps were discarded. Polymorphic sites of the two nuclear genes were phased using a Bayesian statistical method (Stephens and Donnelly, 2003) for haplotype calculation; recombination events detected with DnaSP 5.0 (Rozas et al., 2003) were removed from subsequent analyses.

### Genetic Diversity and Structure

Genetic diversity of *Q. liaotungensis*, such as haplotype diversity (Hd), nucleotide diversity (π) and variance of segregating sites (the Watterson’s estimator per site, 𝜃-W) was calculated for each locus using DnaSP 5.0; a neutrality test using the maximum frequency of derived mutations (MFDM) method (Li, 2011) was conducted to detect selection at each locus with a 5% significance level. Haplotypes were identified in nuclear genes and the concatenated chloroplast region and used to construct networks in PopART using the integer neighbor-joining (NJ) method (Leigh and Bryant, 2015). Haplotype results were visualized on maps with ArcGIS 10.2 (ESRI, Redlands, CA, United States). Analysis of molecular variance (AMOVA) was performed to estimate genetic variation among and within populations, and for phylogenetic groups (see results of population structure) using *F*-statistics with 1000 permutations in Arlequin 3.5 (Excoffier and Lischer, 2010).

The neighbor-net method (NN network) in SplitsTree4 (Bryant and Moulton, 2004) was used to visualize the genetic relationships among all samples. Unphased sequences of the two nuclear genes were concatenated with plastid data to estimate the NN network; the NN method is an extension of the NJ algorithm (Saitou and Nei, 1987) that represents and quantifies conflicting signals and systematic errors among multiple loci. All ambiguous sites were treated by averaging all possible resolutions with Uncorrected *P*-distance and 1000 bootstrap simulations.

Phylogenetic relationships among populations of *Q. liaotungensis* were estimated using the Bayesian method in BEAST 2 (Bouckaert et al., 2014). The Akaike Information Criterion (AIC) with a “greedy” algorithm in PartitionFinder 2.1.1 (Lanfear et al., 2012) was used to select the best-fit partitioning schemes and evolutionary models for the six loci. Based on the AIC results, the dataset was partitioned into five groups (*psb*A-*trn*H, *trn*L-*trn*F + *mat*K, *ycf*1, RPc, and SAP) and phylogenetic relationships were inferred based on the best evolutionary models (Supplementary Table S2) with the coalescent-based Bayesian skyline method and the relaxed clock model in lognormal distribution. Preliminary analyses, assuming all three models of population size (linear, constant, and linear with constant), were run with 5.0 × 10^7^ Markov chain Monte Carlo (MCMC) generations and sampled every 20,000 generations to ensure effective sample size (ESS > 200). Each population size model was performed with four different time seeds and checked for convergence. Molecular dating of divergence among populations and major groups was estimated with selected mutation rates of chloroplast (7.905 ± 0.3 × 10^-10^ per site per year) and nuclear genes (2.20 ± 0.2 × 10^-9^ per site per year) following a previous study (Yang et al., 2016). Four runs of each population size model were combined using LogCombiner. To select the best model that explains population size changes, the three models were compared using Bayes factor (BF) and AIC following MCMC (AICM) methods (Newton and Raftery, 1994; Baele et al., 2012). The best model (linear model, see Supplementary Table S3) was executed with 2.0 × 10^8^ MCMC generations and three independent runs; simulations were combined using LogCombiner. We calculated an unrooted maximum clade credibility (MCC) tree with posterior probability (PP) limit >0.5 using TreeAnnotator of the BEAST 2 package after removing the first 20% of trees as burn-in. The MCC tree was edited and visualized in FigTree 1.4.2.

### Approximate Bayesian Computation

To assess global *Q. liaotungensis* population size changes during the Quaternary, we applied ABC in DIY-ABC 2.1.0 (Cornuet et al., 2014) to seven plausible scenarios. Hierarchical structure (see results of population structure) was ignored because a preliminary analysis, under an isolation-with-migration model with divisions of population structures, did not generate convergent simulations due to limited sampling of each population group. The first three scenarios assumed simple population changes with constant population size (S1), exponential population decline (S2), and exponential population growth (S3). Scenarios 4 and 5 were modifications of S2 and assumed an expansion event before population decline during the Quaternary glacial-interglacial transitions. Considering the complex impact of large effective population sizes and evolutionary time lags in oak species response to environmental changes (Gugger et al., 2013), we assumed either a pre-glacial (from the last interglacial to last glacial maximum, LIG-LGM) (S4) or a post-glacial response (from the LGM to Holocene) (S5) to population decline. By comparison, scenarios 6 and 7 were modifications of scenario 3 and postulated a population decline pre- (LIG to LGM) (S6) or post- (LGM to Holocene) (S7) glacial period before an expansion of population size occurred based on the exponential population change model; each scenario was simulated with a uniform prior probability (Supplementary Figure S1 and Supplementary Table S4). For Scenarios 4 to 7, the current effective population size parameter (Ne) was not constrained against the ancestral population size (Na), as little is known about the ancestral population sizes of *Q. liaotungensis*. Current population sizes were assumed as 10^3^-10^5^ according to a maximum likelihood analysis, while the estimated age of the ancestor was 1-3 Ma across the Pliocene/Pleistocene boundary and the generation time of *Q. liaotungensis* was set at 80 years (Yang et al., 2016).

For the ABC simulation, multiple loci were partitioned into plastid and nuclear datasets. The mutation rates of chloroplast and nuclear genes were the same as those used in the phylogenetic analysis, and the Hasegawa–Kishino–Yano (HKY) mutation model was used for both datasets. We generated a reference table using 7 × 10^6^ simulations and selected five summary statistics (variance of pairwise difference, Tajima’s *D*, private segregating sites, mean of numbers of the rarest nucleotide at segregating sites, and variance of numbers of the rarest nucleotide at segregating sites). To select the most likely model under direct and logistic approaches, we selected 1% of the simulated datasets closest to the observed data to evaluate model accuracy and estimate the relative PP with 95% confidence intervals (95% CI) for each scenario. To estimate type I and II errors on the power of model selection, we assessed confidence in scenario choice with 500 simulated pseudo-observed data sets (PODs) for the seven plausible scenarios. For the best-supported model, performance of all parameter estimations was determined by evaluating the relative median of absolute errors (RMAE) with 500 PODs from the simulated datasets. To assess whether the chosen scenario could explain the observed data correctly under the five summary statistics, we conducted a goodness-of-fit analysis with 1000 PODs of the simulated data by using a principal component analysis (PCA) (Cornuet et al., 2010).

### Ecological Niche Modeling

Occurrence data for *Q. liaotungensis* were retrieved from the Chinese Virtual Herbarium (CVH)^1^. We retained 41 records for ENM analyses after removing duplicate and anomalous sites. For the climatic variables, the following six bioclimatic factors from global climate models (GCMs) were selected due to their relative importance in shaping plant phylogenetic patterns and geographical distribution of *Quercus* (Xu et al., 2013, 2016; Qian and Sandel, 2017): annual mean temperature (BIO1), temperature seasonality (BIO4), mean temperature of coldest quarter (BIO11), annual precipitation (BIO12), precipitation seasonality (BIO15), and precipitation of driest quarter (BIO17). Climatic variables at 2.5 arc-min resolution were obtained from the WorldClim database^2^.

To check for multicollinearity, a Pearson correlation analysis was performed on the six climatic factors, and BIO11 was removed due its strong correlations (*r* > 0.8) with BIO1 and BIO4. Initially, eight simulations of the current geographic distribution of *Q. liaotungensis* were conducted based on present climatic conditions; the regularization multiplier was set from 0.8 to 1.5 and the threshold rule of maximum training sensitivity plus specificity in Maxent v3.3.3k was applied (Phillips et al., 2006). For subsequent simulations, the values of the area under a receiver-operating characteristic curve (AUC) were compared and then an optimal regularization multiplier (1.2) was selected. Suitable current habitats, the LIG (∼120-140 ka), the LGM (∼21 ka) and the mid-Holocene (MH, ∼6 ka) were projected with 10 replicates and 80/20% (train/test) samples for cross-validation. For the LGM and MH periods, we used two different bioclimatic models (Community Climate System Model and Model for Interdisciplinary Research on Climate-Earth System Model, CCSM4 and MIROC-ESM). AUC values of each projected period were used to evaluate model accuracy. We also measured the areas of suitable *Q. liaotungensis* habitat for each period with distributional probability thresholds above 0.6 (moderate) and 0.8 (optimum) in ArcGIS 10.2.

Climatic distributions of the two phylogenetic groups (see results of population structure) were described based on current bioclimatic variables (BIO4 and BIO15) using two dimensional kernel density estimation with the kde2d function of the MASS package (Venables and Ripley, 2002) in R 3.4 (R Core Team, 2011); the two climatic factors showed significantly statistical differentiation (for BIO4, Wilcoxon rank sum test: W = 9.00, *P* = 0.0060, one-way ANOVA: *R*^2^ = 0.2958, *P* = 0.0064; for BIO15, Wilcoxon rank sum test: W = 17.50, *P* = 0.0350, one-way ANOVA: *R*^2^ = 0.2555, *P* = 0.0113) between the two phylogenetic groups.

### Associations Between Genetic Variation and Geographical/Ecological Factors

We used correlation and regression analyses to test whether there was a relationship between genetic variation of two nuclear genes and *Q. liaotungensis* habitat. Chloroplast region genetic data was not used due to relatively low genetic diversity and differentiation. Potential patterns of IBD or IBE among *Q. liaotungensis* populations were assessed with Mantel tests, which were used to evaluate the correlation between genetic and geographical/ecological distances. Pairwise F_ST_ distance calculated in Arlequin 3.5 was used as genetic distance. A matrix of geographical distances among locations was estimated using the Geographic Distance Matrix Generator 1.2.3 (Ersts, 2011). The ecological factors included the five bioclimatic variables used in the ENM and three ecological variables representing soil characteristics (carbon density, soil moisture and soil pH); the latter variables were selected because they seem to play important roles in *Q. liaotungensis* habitat suitability (Li et al., 2012). Ecological distance was calculated using a scaled Euclidean method in PASSaGE v2 (Rosenberg and Anderson, 2011). To estimate the correlations among distance matrices we used a two-tailed test with 999 permutations. Because a significant correlation between geographical and ecological distances (*r* = 0.4005, *P* = 0.0030) was found, a partial Mantel test was also performed to evaluate correlations between genetic differentiation and geographical/ecological distances when one of the abiotic matrices was controlled. All Mantel tests were performed using PASSaGE v2.

Associations of genetic diversity (Hd, π and 𝜃-W) with geographical factors (latitude and longitude) and ecological variables (bioclimatic and soil factors) were assessed using multiple linear regression analysis. Two linear regressions including the generalized linear model (GLM) and the robust linear model (RLM) were used to fit genetic data with each variable and variable combinations. A stepwise method (stepAIC) with forward and backward search was used to estimate the best-fit model based on the AIC algorithm. The two linear models generated a total of 3096 regressions for the geographical factors (36) and ecological variables (3060) in relation to genetic diversity of *Q. liaotungensis*. Variables with the lowest AIC value and significant correlation were retained and considered as appropriate explanatory factors. All linear regressions were performed in R 3.4 using the MASS package.

## Results

### Sequence Information and Genetic Diversity

Six loci were sequenced in 105 individuals from 21 populations of *Q. liaotungensis* (Supplementary Table S1). We detected seven minimum recombination events in the RPc gene and two in the SAP gene. After deleting the recombinant sites, the aligned lengths of the nuclear genes RPc and SAP were 336 and 434 bp, respectively. The aligned chloroplast regions (*psb*A-*trn*H, *trn*L-*trn*F, *ycf*1, and *mat*K) compiled from our previous study were 404, 308, 713, and 389 bp long, respectively. Concatenation of the four chloroplast regions resulted in an 1814 bp alignment with five indels located in the *psb*A-*trn*H (three) and *trn*L-*trn*F (two) regions (Supplementary Table S2). All new sequences were deposited in GenBank (MH232547-MH232601).

Twenty-seven haplotypes were identified in all samples of the phased RPc gene while fourteen haplotypes were revealed in the phased SAP gene. For the RPc gene, the most frequent haplotype H1 was found in all populations and was located toward the center of the network (Figure 1). Eighteen private RPc haplotypes were identified in 10 populations across the distribution range of *Q. liaotungensis*. Population GQ had the highest haplotype diversity (Hd = 0.9333) among the 21 populations for the RPc data (Supplementary Table S1). For the SAP gene, a star-like network suggests that H1 is an ancient haplotype given that it was found in all populations; also, the rest of the SAP haplotypes were derived from H1 through one or two genetic mutations. Furthermore, seven private SAP haplotypes were found in seven populations distributed mainly around the western periphery and central areas of the species distribution range (Figure 1). The highest SAP haplotype diversity was found in the NW population (Hd = 0.7778) (Supplementary Table S1). The concatenated chloroplast region revealed eight haplotypes and each population was represented by one haplotype; of these, five were private haplotypes mainly found in central areas. The low genetic diversity of the chloroplast region may be due, in part, to the limited number of samples used in the present study (Supplementary Figure S2 and Supplementary Table S1).

Compared to the chloroplast data, the RPc and SAP data showed higher haplotype (Hd) and nucleotide diversity (π); also, variance of segregating sites (𝜃-W) in the chloroplast data was lower than in the two nuclear genes. For the nuclear data, the summary statistics revealed higher genetic diversity for the RPc gene than for the SAP gene for all *Q. liaotungensis* populations, as well as for the two genetic clusters (Table 1). No loci significantly deviated from the expectation of neutrality based on neutrality tests with the MFDM method (Table 1).

**Table 1:** Summary statistics of molecular analyses in nuclear and chloroplast loci for *Quercus liaotungensis* and two phylogenetic clusters.

Locus	Group	N	S	Nh	Hd	π	𝜃-W	MFDM
RPc	Species	105	21	27	0.7150	0.0080	0.0105	1.0000
	Cluster 1	75	17	22	0.7400	0.0080	0.0091	0.8859
	Cluster 2	30	15	11	0.6510	0.0079	0.0096	1.0000
SAP	Species	105	14	14	0.3420	0.0009	0.0054	1.0000
	Cluster 1	75	11	11	0.2480	0.0007	0.0045	1.0000
	Cluster 2	30	6	7	0.5370	0.0014	0.0029	1.0000
Chloroplast	Species	105	9	8	0.7280	0.0013	0.0009	0.3883
	Cluster 1	75	4	4	0.5140	0.0005	0.0004	0.2703
	Cluster 2	30	3	4	0.6900	0.0007	0.0004	NA

*N, number of sequences; *S*, number of polymorphic sites; Nh, number of haplotypes; Hd, haplotype diversity; π, nucleotide diversity; 𝜃-W, variance of segregating sites.*

*MFDM, maximum frequency of derived mutations; NA, samples less than 41 were not tested.*

### Genetic Structure and Differentiation

**FIGURE 2:**
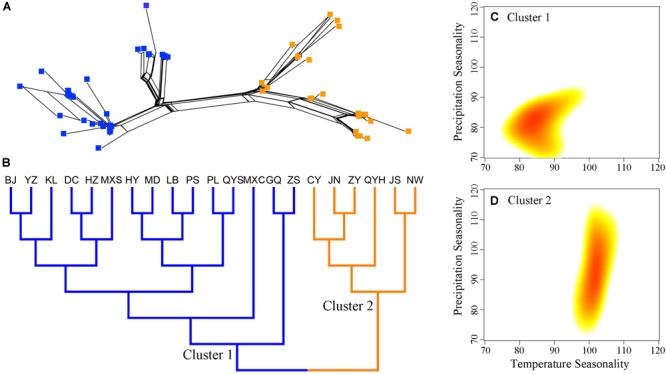
Genetic structure and climatic distributions among populations of *Quercus liaotungensis*. Genetic relationships of individuals **(A)** and populations **(B)** indicate consistent population structures for *Q. liaotungensis*. Climatic distributions of temperature and precipitation seasonality **(C,D)** using two-dimensional kernel density estimation show distinct ecological adaptation among populations from Cluster 1 and Cluster 2. Blue squares and branches represent individuals and populations of Cluster 1, while orange squares and branches indicate individuals and populations from Cluster 2. Red color in climatic distributions indicates climatic regions with the highest density of populations from each cluster.

Genetic clustering of *Q. liaotungensis* populations, based on multiple loci, revealed two highly divergent groups, each with two subclades showing low branch support (Figures 2A,B). A neighbor-net network showed that individuals from populations GQ and ZS in cluster 1, as well as samples from populations QYH and CY in cluster 2 had low genetic distances, thus they were confined to the same area in the network; samples from other populations were mixed together and displayed a complex reticular structure (Supplementary Figure S3).

AMOVA analyses for the SAP gene and chloroplast regions revealed significant differentiation among populations of *Q. liaotungensis* within clusters 1 and 2, as well as between populations in these clusters. However, analyses of the RPc gene did not support significant differentiation within or between clusters (Table 2).

**Table 2:** Analysis of molecular variance (AMOVA) among populations of species and two phylogenetic clusters for *Quercus liaotungensis*.

Locus	Group	Source of variation	Sum of squares	Percentage of variation (%)	*F*_ST/CT_
RPc	Species	Among populations	9.15	3.08	0.0303^∗∗^
		Within populations	65.60	96.92	
	Cluster 1	Among populations	6.54	2.89	0.0289 ns
		Within populations	48.60	97.11	
	Cluster 2	Among populations	2.20	3.82	0.0382 ns
		Within populations	17.00	96.18	
	Cluster 1–Cluster 2	Among groups	0.41	0.17	0.0017 ns
		Within groups	8.74	3.16	
		Within populations	65.60	97.01	
SAP	Species	Among populations	6.20	8.98	0.0898^∗∗∗^
		Within populations	29.50	91.02	
	Cluster 1	Among populations	2.59	5.38	0.0538^∗∗^
		Within populations	15.90	94.62	
	Cluster 2	Among populations	2.25	7.29	0.0729^∗∗^
		Within populations	13.60	92.71	
	Cluster 1–Cluster 2	Among groups	1.36	7.23	0.0723^∗∗^
		Within groups	4.84	5.51	
		Within populations	29.50	87.26	
Chloroplast	Species	Among populations	37.86	100.00	1.0000^∗∗∗^
		Within populations	0.00	0.00	
	Cluster 1	Among populations	19.00	100.00	1.0000^∗∗∗^
		Within populations	0.00	0.00	
	Cluster 2	Among populations	10.00	100.00	1.0000^∗∗∗^
		Within populations	0.00	0.00	
	Cluster 1–Cluster 2	Among groups	8.86	35.91	0.3591^∗∗∗^
		Within groups	29.00	64.09	
		Within populations	0.00	0.00	

*^∗∗∗^*P* < 0.001; ^∗∗^*P* < 0.05; ns, not significant.*

### Demographical History and Effective Population Size

The unrooted evolutionary tree based on multiple loci suggests that the mean estimated divergence time of the two major clusters is 0.29 Ma with 95% highest posterior density (HPD) across 0.10-0.53 Ma. Population diversification in each cluster was almost synchronous and the mean split time of each group was around 0.15 Ma (95% HPD: 0.05-0.30 Ma), while establishment of the present populations began at *ca.* 0.07 Ma (95% HPD: 0.02-0.17 Ma) (Supplementary Figure S4).

**FIGURE 3:**
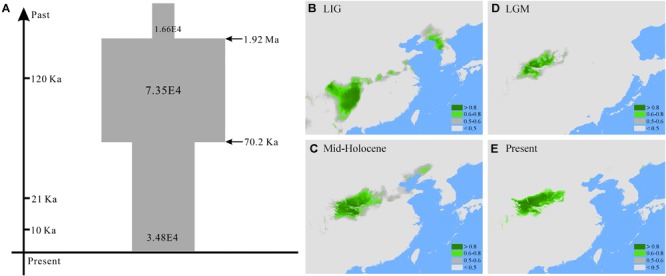
**(A)** Inferred demographical history of *Quercus liaotungensis* from the best simulated model (scenario 4) in approximate Bayesian computation and potential habitats of *Q. liaotungensis* during the **(B)** last interglacial, **(C)** the last glacial maximum, **(D)** the mid-Holocene, and **(E)** present estimated with ecological niche modeling.

Based on multiple loci, ABC analyses revealed that scenario 4 provided the best fit model to explain *Q. liaotungensis* global population changes under direct estimate (0.2099, 95% CI: 0.1798-0.2401) and logistic regression tests (0.2275, 95% CI: 0.2131-0.2419) (Supplementary Figure S5); furthermore, 95% CI of the PP for this scenario did not overlap with other scenarios (except for the second best supported scenario 5) under logistic estimation (Supplementary Table S4). Model checking analysis of ABC simulations showed that the summary statistics from the observed data produced eigenvectors that were within or at the margins of 1000 simulated PODs from the posterior distribution for all seven plausible scenarios (Supplementary Figure S6). Also, most of the summary statistics were not significantly different when the observed and simulated data (*P* value > 0.1) for the best-supported scenario 4 were compared, suggesting reliable results for the ABC simulation. For the best supported scenario 4, power analyses of confidence scenario choice showed low values of type I (direct estimate: 0.096; logistic estimate: 0.110) and type II errors (direct estimate: 0.084; logistic estimate: 0.093) based on 500 PODs; the RMAE values for all parameters of scenario 4 were moderately low (Table 3), indicating reliable estimates of posterior parameters from our ABC simulations. Lower support was found for scenario 5 (direct estimate: 0.2061; logistic estimate: 0.2072), which also assumed an expansion decline trend for the population dynamics of *Q. liaotungensis* as revealed by scenario 4, while the rest scenarios had relatively low posterior supports (PP < 0.2) (Supplementary Figure S5). Given the assumption of 80 years for the generation time of *Q. liaotungensis*, the best fit model (S4) of the ABC simulation indicated that the ancestor of *Q. liaotungensis* could, at least date back to *ca.* 1.92 Ma (95% HPD: 1.09-2.87 Ma) with a relatively low population size (16,600). Under these conditions, the effective population size of the ancestral population expanded by *ca.* five times (73,500) from 1.92 Ma until 70.2 ka (95% HPD: 25-115 ka), and was later followed by a population decline to nearly half the largest population size (34,800), which has continued up to the present (Figure 3A and Table 3).

**Table 3:** Prior settings and estimations of posterior distributions of parameters for the best supported scenario (S4) of demographical history of *Quercus liaotungensis* with approximate Bayesian computation.

Parameter	Prior value	Mean	Median	Mode	95% low	95% high	RMAE
Ne	1E3∼1E5	3.48E + 04	3.29E + 04	2.87E + 04	1.69E + 04	5.98E + 04	0.204
Ta (year)	1.25E4∼3.75E4	1.92E + 06	1.88E + 06	1.22E + 06	1.09E + 06	2.87E + 06	0.217
Tdb (year)	2.5E2∼1.5E3	7.02E + 04	7.02E + 04	9.60E + 04	2.50E + 04	1.15E + 05	0.297
Ndb	1E3∼1E5	7.35E + 04	7.63E + 04	9.60E + 04	3.97E + 04	9.78E + 04	0.143
Na	1E3∼1E5	1.66E + 04	1.19E + 04	1.69E + 03	1.81E + 03	4.82E + 04	0.373
μcpDNA	5E-8∼7E-8	5.93E-08	5.92E-08	5.00E-08	5.07E-08	6.85E-08	0.089
μnrDNA	1E-7∼3E-7	1.78E-07	1.70E-07	1.26E-07	1.07E-07	2.74E-07	0.181

*Ne, current effective population size; Na, ancestral effective population size; Ndb, effective population size after expansion or decline; Ta, time of ancestral effective population; Tdb, time of effective population changes; μ, mutation rate of genetic loci; RMAE, relative median absolute error.*

### Ecological Niche Modeling and Climatic Distribution

The high AUC values for each period indicate accurate simulations of the ecological niche models for *Q. liaotungensis*. For the LGM and MH periods, the CCSM4 and MIROC-ESM models showed consistent AUC values (Supplementary Table S5). Prediction for the LIG period showed that *Q. liaotungensis* occurred at low latitudes (around 25-30°N) and occupied the largest suitable habitat areas compared to LGM, MH and the present. During the LGM, the species moved northward to the Qinling Mountains and its distribution range decreased, consistent with the shrinkage of suitable habitat areas. The predicted distribution of *Q. liaotungensis* during the MH and the present suggest that this species had a relatively stable habitat after the LGM; however, the estimated habitat areas increased from the LGM to the MH. Also, the areas of current habitat are slightly smaller than those estimated for the MH (Figures 3B–E and Supplementary Table S5).

Statistical analyses suggest that the two *Q. liaotungensis* phylogenetic clusters have significant differences in ecological adaptation to temperature seasonality (BIO4) and precipitation seasonality (BIO15). Populations in cluster 1 seem to have adapted to low levels of temperature and precipitation seasonality, while populations in cluster 2 might have adapted to habitats with higher temperature seasonality and broader niche breadth of precipitation seasonality (Figures 2C,D).

### Associations Between Genetic Variation and Geographical/Ecological Factors

The results of Mantel tests based on SAP gene data showed a significant and positive correlation (*r* = 0.3057, *P* = 0.0280) between genetic and ecological distance for *Q. liaotungensis*; interestingly, when geographical distance was controlled using a partial Mantel test, the correlation between genetic differentiation and ecological distance was enhanced (*r* = 0.3245, *P* = 0.0310). Separate analysis of SAP data for cluster 1 revealed marginal significant correlations between genetic and ecological distances when Mantel and partial Mantel tests were conducted; by contrast, no significant correlations were found for cluster 2. Similarly, the RPc data showed no correlations between genetic and ecological/geographical distances for clusters 1 and 2 (Table 4).

**Table 4:** Results of Mantel tests between genetic (GeD) and geographical (GD), and genetic (GeD) and ecological (ED) distances for *Quercus liaotungensis* and two phylogenetic clusters based on two nuclear data.

		RPc		SAP	
Group	Matrix	*r*	*P*	*r*	*P*
Species	GeD-GD	0.0201	0.8730	0.0212	0.8670
	GeD-ED	0.1737	0.1840	0.3057	0.0280
	GeD-GD (ED partial)	–0.0871	0.4710	–0.1160	0.3130
	GeD-ED (GD partial)	0.1927	0.1420	0.3245	0.0310
Cluster 1	GeD-GD	–0.1216	0.4290	0.1354	0.3540
	GeD-ED	0.0315	0.8320	0.4288	0.0550
	GeD-GD (ED partial)	–0.1511	0.3170	–0.0661	0.6960
	GeD-ED (GD partial)	0.0956	0.5270	0.4151	0.0520
Cluster 2	GeD-GD	–0.1443	0.7130	–0.3320	0.2330
	GeD-ED	0.1463	0.6420	–0.2303	0.4250
	GeD-GD (ED partial)	–0.2887	0.3490	–0.2698	0.4050
	GeD-ED (GD partial)	0.2896	0.3280	–0.1150	0.6620

**FIGURE 4:**
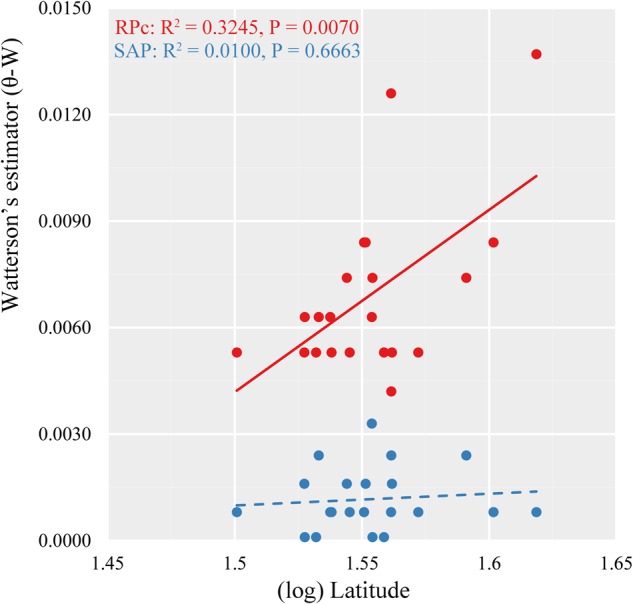
Relationship between variance of segregating sites (Watterson’s estimator, 𝜃-W) and latitude in 21 locations of *Quercus liaotungensis* based on two nuclear genes.

**FIGURE 5:**
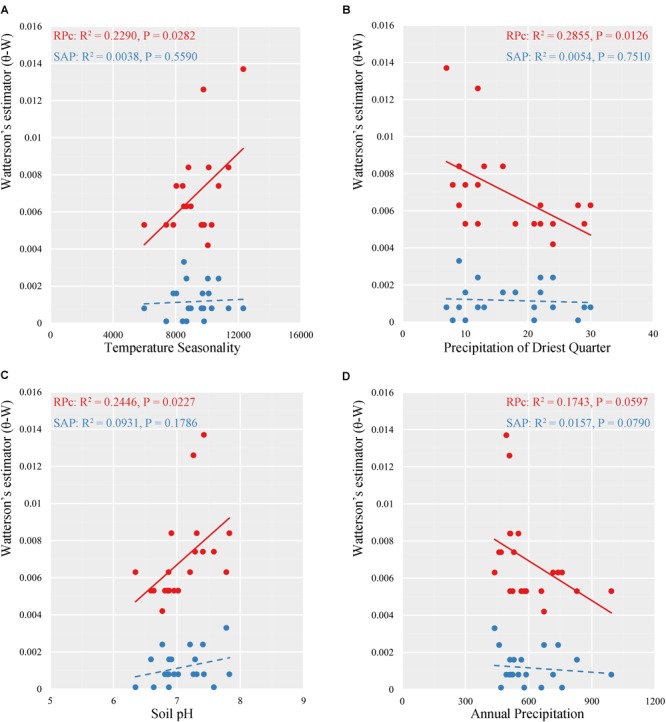
Linear regressions for relationships between variance of segregating sites (𝜃-W) and four ecological variables (**A**, temperature seasonality; **B** precipitation of driest quarter; **C**, soil pH; **D**, annual precipitation) in two nuclear data for *Quercus liaotungensis*.

Multiple linear regressions indicated consistent results between the GLM and the RLM simulations based on stepAIC estimation (Supplementary Table S6). Based on RPc data, GLM regressions revealed significant correlations between distribution of genetic diversity and abiotic factors. Specifically, (log) latitude showed significant positive correlation with variance of segregating sites (𝜃-W) (*R*^2^ = 0.3245, *P* = 0.0070) for the 21 locations tested (Figure 4). Of the eight ecological factors evaluated, the optimum model selected with the AIC method suggested a combination of five variables (BIO4 + BIO12 + BIO15 + BIO17 + soil pH) (AIC = -201.22, *R*^2^ = 0.6227, *P* = 0.0071) to explain the differentiation of 𝜃-W among *Q. liaotungensis* populations (Supplementary Table S6). Regressions on each of these five ecological factors indicated that 𝜃-W showed significant positive relationships with BIO4 (temperature seasonality) (*R*^2^ = 0.2290, *P* = 0.0282) and soil pH (*R*^2^ = 0.2446, *P* = 0.0227), and a negative correlation with BIO17 (precipitation of driest quarter) (*R*^2^ = 0.2855, *P* = 0.0126) (Figures 5A,C). Moreover, a marginally significant and negative relationship was observed between 𝜃-W and BIO12 (annual precipitation) (Figure 5D). By comparison, no significant associations were found among all regression models for the SAP gene (Supplementary Table S6), suggesting that none of abiotic factors tested could provide a good explanation for the distribution of the genetic diversity of this gene.

## Discussion

### Demographical History of *Q. liaotungensis*

Using chloroplast and nuclear data from 21 populations and ABC simulations, we estimate that under the best supported scenario 4 the most recent common ancestor of *Q. liaotungensis* could date back, at least, to the early Pleistocene (1.92 Ma, 95% HPD: 1.09-2.87 Ma) (Figure 3A and Table 3); this possible recent speciation event for *Q. liaotungensis* is highly consistent with our previous research using an isolation-with-migration model for *Q. liaotungensis* and *Q. mongolica* (Yang et al., 2016), and with the proximity to the Largest Glaciation at *ca.* 0.6-1.2 Ma and the deformed uplift of the Qinghai-Tibetan Plateau (QTP) during *ca.* 1.3-3 Ma (Zheng et al., 2002; Sun et al., 2015). Furthermore, recent studies also suggest that Pleistocene climatic fluctuations played an important role triggering plant speciation in members of *Roscoea* in the Hengduan Mountain regions, as well as in some closely related tree species with long generation times in the genera *Quercus, Populus*, and *Juglans* (Levsen et al., 2012; Zhao et al., 2016; Leroy et al., 2017; Bai et al., 2018).

Coalescent-based ABC analyses of multiple loci indicate a recent population expansion of *Q. liaotungensis*, which seems to have been predated by a small effective population size (Figure 3A); this expansion possibly began in the early Pleistocene around 1.92 Ma (Figure 3A and Table 3). This increase in effective population sizes could have been the result of rapid adaptation of *Q. liaotungensis* to glacial cycles during the Pleistocene *via* genetic differentiation and/or local adaptation. Support for this scenario comes from the fact that *Q. liaotungensis* populations are genetically differentiated into two major clusters with a deep divergence at *ca.* 0.29 Ma according to the unrooted Bayesian evolutionary tree (Supplementary Figure S4). Furthermore, considering the niche conservatism of recently diverged lineages (Wiens, 2004), the distinct climatic distributions (in terms of temperature and precipitation seasonality, Figures 2C,D) of these two clusters may indicate rapid adaptation of this oak species to different climatic elements during an early stage of intraspecific differentiation (Hipp et al., 2018). Estimates of divergence time for clusters 1 and 2 suggest that ancestral populations of each cluster were initially differentiated during *ca.* 150-130 ka, followed by synchronously increased population diversification from ∼70 ka to present (Supplementary Figure S4). At the same time, the best supported scenario 4 of ABC simulations revealed a decline in the effective population size of *Q. liaotungensis* from this time period (70.2 ka) to present time (Figure 3A), corresponding closely with a rapid increase in global ice volume of Marine Isotope Stage 4 (∼76 ka) (Landais, 2004) and the Baiyu Glaciation (70-10 ka) recognized on the QTP during the Quaternary glacial cycles (Zheng et al., 2002). This result is further supported by ENM simulations, which show that suitable habitat areas were remarkably small during the LGM in contrast to the LIG period (Figures 3B,D and Supplementary Table S5) when ancestral populations of each phylogenetic cluster were initially differentiated (Supplementary Figure S4). Thus, it is plausible that glaciation events from ∼70 ka had a severe impact on *Q. liaotungensis* resulting in decreased population sizes and isolation. Moreover, during this time period, intraspecific genetic differentiation could have been enhanced by limited gene flow among isolated populations (Hewitt, 2004).

Different lines of evidence from the ABC simulations and ENMs suggest a trend of expansion-decline of *Q. liaotungensis* populations during the Quaternary. However, a caveat of the estimated time of effective population changes is the relatively low posterior probabilities obtained for the models compared in the ABC simulations (Supplementary Figure S5 and Supplementary Table S4). Furthermore, to explain the demography of this oak species, we only considered seven simple scenarios with global population changes during the ABC analyses (Supplementary Figure S1 and Supplementary Table S4); also, the genetic structure of populations was not simulated due to small sample size and insufficient genetic information of the individuals in each phylogenetic cluster. Considering the potential variability of responses to climate change and environmental disturbance across intraspecific levels (Maguire et al., 2018), the two genetic clusters of *Q. liaotungensis* (Figure 2) might be the result of differences in spatial and temporal responses during the glacial-interglacial transitions of the Quaternary. Moreover, gene flow between the two phylogenetic clusters and closely related species (e.g., *Q. mongolica*, Zeng et al., 2011) were not accounted for in our ABC simulation; all of these factors could lead to an underestimation of effective population sizes and time of population changes for *Q. liaotungensis*. For ENM analyses, model projections of the four time periods only indicated discrete population distributions of *Q. liaotungensis* since the LIG (Figures 3B–E). However, a plausible scenario of rapid adaptation with population expansion after establishment of the oak species (Figure 3A) was not confirmed by the ENMs due to the lack of ecological niche models during the early Pleistocene (around 1.92 Ma). Thus, further work with sufficient genomic data and available bioclimatic models is necessary to improve our understanding on the intraspecific divergence and demography of *Q. liaotungensis*.

### Associations Between Genetic Variation and Abiotic Factors

Ecological niche modeling projections suggest that suitable habitats for *Q. liaotungensis* have remained relatively stable after the LGM (Figures 3C–E). Furthermore, ENMs and genetic results (Figures 1, 3B–E, Supplementary Figure S2, and Supplementary Table S1) indicate that central areas of *Q. liaotungensis*’ current distribution range, such as the Qinling Mountains and Taihang Mountains, could have been potential refugia during glacial periods (Zeng et al., 2011; Yang et al., 2016). The ENMs showed that this species contracted toward the central regions (e.g., Qinling Mountains) of its distribution range during the LGM and then increased its distribution when temperature ascended after the glacial period (Figures 3C–E and Supplementary Table S5). Under these circumstances, a possible outcome could be increased genetic differentiation between central and peripheral populations, as well as low genetic diversity of peripheral populations due to the center-peripheral effect (Ortego et al., 2012). However, association analyses did not support the refuge model because the RPc data showed a significant positive correlation between the variance of segregating sites (𝜃-W) and latitude (Figure 4). Moreover, Mantel tests based on the two nuclear genes did not detect IBD patterns, suggesting that the spatial genetic structure of *Q. liaotungensis* is independent of geographical distance among locations (Table 4). Also, it is possible that intraspecific population structure (Figure 2) may have been erased via efficient and long-distance gene flow during gradual expansion and migration (Moyle, 2006). In addition, interspecific hybridization could have impacted our association analyses in spite of our rigorous sampling strategy to exclude hybrids and introgressants. In general, hybridization would add more complexity to the distribution of genetic variation across the species distribution range.

Multiple linear regression analyses of RPc data showed significant relationships between variance of segregating sites (𝜃-W) and temperature seasonality, and precipitation of the driest quarter (Figure 5 and Supplementary Table S6). By contrast, the SAP data did not support a significant correlation between these variables; however, Mantel tests with SAP data showed significant correlations between genetic differentiation and ecological distances (Table 4).

Regarding neutrality tests, the MFDM results indicated that the two nuclear genes and the concatenated chloroplast region showed no significant deviation from neutrality (Table 1). Given that genetic variation of neutral genes may be associated with rapid climatic changes due to genetic linkage and/or recombination with loci under selection through divergence hitchhiking (Via and West, 2008), it is plausible that Quaternary climatic fluctuations left deep signatures on the genetic differentiation of *Q. liaotungensis* and that the adaptive differentiation among populations could be mirrored by the neutral loci used in this study. However, as neutral markers are less affected by intraspecific gene flow during ecological divergence, this hypothesis will require further investigation using markers from divergently selected genomic regions (Via and West, 2008).

Climate seasonality, as a prevailing condition that shapes organisms’ phenology (e.g., flowering time and growing season), is a prerequisite for stabilization of ecological systems (Quintero et al., 2014). Our results suggest that the two main phylogenetic groups split prior to the LGM and have adapted to distinct ecological niches with unique temperature and precipitation seasonality resulting in high genetic differentiation (Figures 2, Supplementary Figure S4, and Table 2). Also, these two phylogenetic clusters seem to be less correlated with the geographical location of the sampled populations. This result is different from the population genetic structure found in a previous research using neutral genetic markers (Zeng et al., 2011). A possible explanation is that functional genomic regions might display different introgression patterns in contrast to the neutral loci, and reinforce ecological divergence among closely related oaks, even in a contact zone (Leroy et al., 2017). Moreover, it is possible that gene flow between populations of clusters 1 and 2 is limited due to potential reproductive isolation caused by asynchronous phenology; however, testing of this scenario will require further studies using sufficient genomic data and phenological observations. Nevertheless, the aforementioned results support the scenario that unstable regions with moderate levels of environmental disturbance (climate seasonality) effectively impact the genetic differentiation and turnover among populations (the asynchrony of seasons hypothesis) (Martin et al., 2009). A similar situation was detected in the Californian canyon live oak (*Quercus chrysolepis*), suggesting that habitat instability mediated by climate seasonality is a major factor in driving historical range shifts and shaping genetic variation of tree species having long distance gene flow (Ortego et al., 2015; Bemmels et al., 2016).

A previous study using variation partitioning simulations indicated that precipitation is one of the most important environmental factors determining the geographical ranges of *Q. liaotungensis* (Li et al., 2012). Similarly, based on RPc data, our results also predict a significant negative correlation between genetic diversity (𝜃-W) and precipitation of the driest quarter (*P* = 0.0126), and suggest a marginally significant negative correlation of 𝜃-W with annual precipitation (*P* = 0.0597) (Figure 5), possibly reflecting a drought-tolerance characteristic of *Q. liaotungensis* populations. This pattern appears to be supported by population GQ, which is located at the center of the Loess Plateau, where extreme and arid climate may have induced local adaptation resulting in high levels of genetic diversity and the presence of private haplotypes (Figure 1, Supplementary Figure S2, and Supplementary Table S1). Additionally, soil conditions may influence plant growth and establishment through their effect on the activity of symbiotic microorganisms, which may drive genetic differences among populations (Figure 5C) as well as shape the demographical history of plant species by coevolutionary interactions (Piculell et al., 2008; Bai et al., 2018; Hipp et al., 2018).

## Conclusion

This investigation explores the demographical history and genetic variation of *Q. liaotungensis* in the face of climate changes during the Quaternary using multiple loci and coalescent-based simulations and ENMs. Our results suggest that climatic oscillations during the Pleistocene have greatly affected the effective population sizes and genetic structure of *Q. liaotungensis*. Habitat stability can maintain species genetic diversity but might not act as a direct factor in generating population differentiation of this oak during the glacial-interglacial periods. By comparison, ecological factors, such as the variation of climate seasonality and soil conditions seem to be more important in shaping the genetic structure and genetic diversity of *Q. liaotungensis*. Based on these results, our study highlights the effects of past and present ecological factors on the historical population dynamics and temporal genetic properties of tree species in North China.

## Author Contributions

JY and GZ conceived the research. JY, LF, and ZL collected the samples. JY, LV, LF, and ZL analyzed the data. JY, LV, and GZ wrote the manuscript. All authors read and approved the final manuscript.

## Conflict of Interest Statement

The authors declare that the research was conducted in the absence of any commercial or financial relationships that could be construed as a potential conflict of interest.

## Footnotes

1 http://www.cvh.ac.cn

2 http://www.worldclim.org

## Supplementary Material

FIGURE S1 Scenarios tested using approximate Bayesian computation (ABC) for global changes in population sizes of *Q. liaotungensis*. Na, ancestral population size; Ne, current population size; Ndb, population changes between Na and Ne; Tdb, transfer time between Ndb and Ne; Ta, established time of ancestral populations.

FIGURE S2 Haplotype distributions and network of concatenated chloroplast regions for *Quercus liaotungensis* in Northern China. Pie charts are proportional to the numbers of samples in each population (five individuals per site), and different pie colors correspond to different haplotypes in the network.

FIGURE S3 Detailed neighbor-net network of all individuals indicates genetic relationships of *Quercus liaotungensis*. Blue and orange squares represent samples identified in clusters 1 and 2, respectively. Bootstrap values >50 are shown near the branch.

FIGURE S4 Time-estimated phylogenetic tree of 21 populations from *Quercus liaotungensis* reveals two major clusters using coalescent-based Bayesian analysis. Branches in blue and orange indicate populations belonging to phylogenetic clusters 1 and 2, respectively. Values of posterior probability >0.6 are shown on the branch.

FIGURE S5 Comparison of posterior probabilities of seven simulated scenarios obtained with direct estimate and logistic regression from 1% of closest datasets for approximate Bayesian computation.

FIGURE S6 Panels of principal component analysis along the first three axes for five summary statistics of seven simulated scenarios for model checking in approximate Bayesian computation.

## References

[B1] Baele G. Lemey P. Bedford T. Rambaut A. Suchard M. A. Alekseyenko A. V. (2012). Improving the accuracy of demographic and molecular clock model comparison while accommodating phylogenetic uncertainty. *Mol. Biol. Evol.* 29 2157–2167. 10.1093/molbev/mss084 22403239 PMC3424409

[B2] Bagnoli F. Tsuda Y. Fineschi S. Bruschi P. Magri D. Zhelev P. (2016). Combining molecular and fossil data to infer demographic history of *Quercus cerris*: insights on European eastern glacial refugia. *J. Biogeogr.* 43 679–690. 10.1111/jbi.12673

[B3] Bai W. N. Liao W. J. Zhang D. Y. (2010). Nuclear and chloroplast DNA phylogeography reveal two refuge areas with asymmetrical gene flow in a temperate walnut tree from East Asia. *New Phytol.* 188 892–901. 10.1111/j.1469-8137.2010.03407.x 20723077

[B4] Bai W. N. Yan P. C. Zhang B. W. Woeste K. E. Lin K. Zhang D. Y. (2018). Demographically idiosyncratic responses to climate change and rapid Pleistocene diversification of the walnut genus *Juglans* (Juglandaceae) revealed by whole-genome sequences. *New Phytol.* 217 1726–1736. 10.1111/nph.14917 29178135

[B5] Bemmels J. B. Title P. O. Ortego J. Knowles L. L. (2016). Tests of species-specific models reveal the importance of drought in postglacial ranges shifts of a Mediterranean-climate tree: insights from integrative distributional, demographic and coalescent modellong and ABC model selection. *Mol. Ecol.* 25 4889–4906. 10.1111/mec.13804 27540890

[B6] Bennett K. D. Provan J. (2008). What do we mean by ’refugia’? *Quat. Sci. Rev.* 27 2449–2455. 10.1016/j.quascirev.2008.08.019

[B7] Bouckaert R. Heled J. Kuhnert D. Vaughan T. Wu C. H. Xie D. (2014). BEAST 2: a software platform for Bayesian evolutionary analysis. *PLoS Comput. Biol.* 10:e1003537. 10.1371/journal.pcbi.1003537 24722319 PMC3985171

[B8] Bryant D. Moulton V. (2004). Neighbor-net: an agglomerative method for the construction of phylogenetic networks. *Mol. Biol. Evol.* 21 255–265. 10.1093/molbev/msh018 14660700

[B9] Chen K. M. Abbott R. J. Milne R. I. Tian X. M. Liu J. Q. (2008). Phylogeography of *Pinus tabulaeformis* Carr. (Pinaceae), a dominant species of coniferous forest in northern China. *Mol. Ecol.* 17 4276–4288. 10.1111/j.1365-294X.2008.03911.x 19378405

[B10] Cornuet J. M. Pudlo P. Veyssier J. Dehne-Garcia A. Gautier M. Leblois R. (2014). DIYABC v2.0: a software to make approximate Bayesian computation inferences about population history using single nucleotide polymorphism, DNA sequence and microsatellite data. *Bioinformatics* 30 1187–1189. 10.1093/bioinformatics/btt763 24389659

[B11] Cornuet J. M. Ravigne V. Estoup A. (2010). Inference on population history and model checking using DNA sequence and microsatellite data with the software DIYABC (v1.0). *BMC Bioinformatics* 11:401. 10.1186/1471-2105-11-401 20667077 PMC2919520

[B12] Du S. Yamanaka N. Yamamoto F. Otsuki K. Wang S. Q. Hou Q. C. (2007). The effect of climate on radial growth of *Quercus liaotungensis* forest trees in Loess Plateau, China. *Dendrochronologia* 25 29–36. 10.1016/j.dendro.2007.01.005

[B13] Elias M. Faria R. Gompert Z. Hendry A. (2012). Factors influencing progress toward ecological speciation. *Int. J. Ecol.* 2012 1–7. 10.1155/2012/235010 19127610

[B14] Ersts P. J. (2011). *Geographic Distance Matrix Generator (version 1.2.3). American Museum of Natural History, Center for Biodiversity and Conservation.* Available at: http://biodiversityinformatics.amnh.org/open_sour

[B15] Excoffier L. Lischer H. E. L. (2010). Arlequin suite ver 3.5: a new series of programs to perform population genetics analyses under Linux and Windows. *Mol. Ecol. Resour.* 10 564–567. 10.1111/j.1755-0998.2010.02847.x 21565059

[B16] Gugger P. F. Ikegami M. Sork V. L. (2013). Influence of late Quaternary climate change on present patterns of genetic variation in valley oak, *Quercus lobata* Nee. *Mol. Ecol.* 22 3598–3612. 10.1111/mec.12317 23802553

[B17] Hall T. A. (1999). BioEdit: a user-friendly biological sequence alignment editor and analysis program for Windows 95/98/NT. *Nucleic Acids Symp. Ser.* 41 95–98.

[B18] Harrison S. P. Yu G. Takahara H. Prentice I. C. (2001). Palaeovegetation. Diversity of temperate plants in east Asia. *Nature* 413 129–130. 10.1038/35093169 11557970

[B19] He Q. Edwards D. L. Knowles L. L. (2013). Integrative testing of how environments from the past to the present shape genetic structure across landscapes. *Evolution* 67 3386–3402. 10.1111/evo.12159 24299395

[B20] Hewitt G. M. (2000). The genetic legacy of the Quaternary ice ages. *Nature* 405 907–913. 10.1038/35016000 10879524

[B21] Hewitt G. M. (2004). Genetic consequences of climatic oscillations in the Quaternary. *Philos. Trans. R. Soc. Lond. B Biol. Sci.* 359 183–195. 10.1098/rstb.2003.1388 15101575 PMC1693318

[B22] Hipp A. L. Manos P. S. Gonzalez-Rodriguez A. Hahn M. Kaproth M. McVay J. D. (2018). Sympartic parallel diversification of major oak clades in the Americas and the origins of Mexican species diversity. *New Phytol.* 217 439–452. 10.1111/nph.14773 28921530

[B23] Kremer A. Ronce O. Robledo-Arnuncio J. J. Guillaume F. Bohrer G. Nathan R. (2012). Long-distance gene flow and adaptation of forest trees to rapid climate change. *Ecol. Lett.* 15 378–392. 10.1111/j.1461-0248.2012.01746.x 22372546 PMC3490371

[B24] Landais A. (2004). A continuous record of temperature evolution over a sequence of Dansgaard-Oeschger events during Marine Isotopic Stage 4 (76 to 62 kyr BP). *Geophys. Res. Lett.* 31:L22211 10.1029/2004GL021193

[B25] Landguth E. L. Cushman S. A. Schwartz M. K. McKelvey K. S. Murphy M. Luikart G. (2010). Quantifying the lag time to detect barriers in landscape genetics. *Mol. Ecol.* 19 4179–4191. 10.1111/j.1365-294X.2010.04808.x 20819159

[B26] Lanfear R. Calcott B. Ho S. Y. Guindon S. (2012). Partitionfinder: combined selection of partitioning schemes and substitution models for phylogenetic analyses. *Mol. Biol. Evol.* 29 1695–1701. 10.1093/molbev/mss020 22319168

[B27] Leigh J. W. Bryant D. (2015). popart: full-feature software for haplotype network construction. *Methods Ecol. Evol.* 6 1110–1116. 10.1111/2041-210X.12410

[B28] Leroy T. Roux C. Villate L. Bodenes C. Romiguier J. Paiva J. A. (2017). Extensive recent secondary contacts between four European white oak species. *New Phytol.* 214 865–878. 10.1111/nph.14413 28085203 PMC5624484

[B29] Levsen N. D. Tiffiin P. Olson M. S. (2012). Pleistocene speciation in the genus Populus (Salicaceae). *Syst. Biol.* 61 401–412. 10.1093/sysbio/syr120 22213709 PMC3529545

[B30] Li G. Q. Liu C. C. Liu Y. G. Yang J. Zhang X. S. Guo K. (2012). Effects of climate, disturbance and soil factors on the potential distribution of Liaotung oak (*Quercus wutaishanica* Mayr) in China. *Ecol. Res.* 27 427–436. 10.1007/s11284-011-0914-4

[B31] Li H. P. (2011). A new test for detecting recent positive selection that is free from the confounding impacts of demography. *Mol. Biol. Evol.* 28 365–375. 10.1093/molbev/msq211 20709734

[B32] Liu J. Q. Sun Y. S. Ge X. J. Gao L. M. Qiu Y. X. (2012). Phylogeographic studies of plants in China: advances in the past and directions in the future. *J. Syst. Evol.* 50 267–275. 10.1111/j.1759-6831.2012.00214.x

[B33] Maguire K. C. Shinneman D. J. Potter K. M. Hipkins V. D. (2018). Intraspecific niche models for Ponderosa pine (*Pinus ponderosa*) suggest potential variability in population-level response to climate change. *Syst. Biol.* 67 965–978. 10.1093/sysbio/syy017 29548012

[B34] Martin P. R. Bonier F. Moore I. T. Tewksbury J. J. (2009). Latitudinal variation in the asynchrony of seasons implications for higher rates of population differentiation and speciation in the tropics. *Ideas Ecol. Evol.* 2 9–17. 10.4033/iee.2009.2.3.n

[B35] Moyle L. C. (2006). Correlates of genetic differentiation and isolation by distance in 17 congeneric Silene species. *Mol. Ecol.* 15 1067–1081. 10.1111/j.1365-294X.2006.02840.x 16599967

[B36] Newton M. A. Raftery A. E. (1994). Approximate Bayesian inference with the weighted likelihood bootstrap. *J. R. Stat. Soc. Ser. B* 56 3–48.

[B37] Orsini L. Vanverbeke J. Swillen I. Mergeay J. Meester L. D. (2013). Drivers of population genetic differentiation in the wild: isolation by dispersal limitation, isolation by adaptation and isolation by colonization. *Mol. Ecol.* 22 5983–5999. 10.1111/mec.12561 24128305

[B38] Ortego J. Gugger P. F. Sork V. L. (2015). Climatically stable landscapes predict patterns of genetic structure and admixture in the Californian canyon live oak. *J. Biogeogr.* 42 328–338. 10.1111/jbi.12419

[B39] Ortego J. Riordan E. C. Gugger P. F. Sork V. L. (2012). Influence of environmental heterogeneity on genetic diversity and structure in an endemic southern Californian oak. *Mol. Ecol.* 21 3210–3223. 10.1111/j.1365-294X.2012.05591.x 22548448

[B40] Petit R. J. Csaikl U. M. Bordacs S. Burg K. Coart E. Cottrell J. (2002). Chloroplast DNA variation in European white oaks phylogeography and patterns of diversity based on data from over 2600 populations. *For. Ecol. Manage.* 156 5–26. 10.1016/S0378-1127(01)00645-4

[B41] Phillips S. J. Anderson R. P. Schapire R. E. (2006). Maximum entropy modeling of species geographic distributions. *Ecol. Modell.* 190 231–259. 10.1016/j.ecolmodel.2005.03.026

[B42] Piculell B. J. Hoeksema J. D. Thompson J. N. (2008). Interactions of biotic and abiotic environmental factors in an ectomycorrhizal symbiosis, and the potential for selection mosaics. *BMC Biol.* 6:23. 10.1186/1741-7007-6-23 18507825 PMC2430191

[B43] Qian H. Ricklefs R. E. (2000). Large-scale processes and the Asian bias in species diversity of temperate plants. *Nature* 407 180–182. 10.1038/35025052 11001054

[B44] Qian H. Sandel B. (2017). Phylogenetic structure of regional angiosperm assemblages across latitudinal and climatic gradients in North America. *Glob. Ecol. Biogeogr.* 26 1258–1269. 10.1111/geb.12634

[B45] Quintero I. Gonzalez-Caro S. Zalamea P. C. Cadena C. D. (2014). Asynchrony of seasons genetic differentiation associated with geographic variation in climatic seasonality and reproductive phenology. *Am. Nat.* 184 352–363. 10.1086/677261 25141144

[B46] R Core Team (2011). *R: A Language and Environment for Statistical Computing.* Vienna: R Foundation for Statistical Computing.

[B47] Ribera I. Castro A. Díaz J. A. Garrido J. Izquierdo A. Jäch M. A. (2011). The geography of speciation in narrow-range endemics of the ‘Haenydra’ lineage (Coleoptera, Hydraenidae, Hydraena). *J. Biogeogr.* 38 502–516. 10.1111/j.1365-2699.2010.02417.x

[B48] Rosenberg M. S. Anderson C. D. (2011). PASSaGE: pattern analysis, spatial statistics and geographic exegesis. version 2. *Methods Ecol. Evol.* 2 229–232. 10.1111/j.2041-210X.2010.00081.x

[B49] Rozas J. Sanchez-Delbarrio J. C. Messeguer X. Rozas R. (2003). DnaSP, DNA polymorphism analyses by the coalescent and other methods. *Bioinformatics* 19 2496–2497. 10.1093/bioinformatics/btg359 14668244

[B50] Saitou N. Nei M. (1987). The Neighbor-joining method: a new method for reconstructing phylogenetic trees. *Mol. Biol. Evol.* 4 406–425. 10.1093/oxfordjournals.molbev.a040454 3447015

[B51] Stephens M. Donnelly P. (2003). A comparison of Bayesian methods for haplotype reconstruction from population genotype data. *Am. J. Hum. Genet.* 73 1162–1169. 10.1086/379378 14574645 PMC1180495

[B52] Stewart J. R. Lister A. M. Barnes I. Dalen L. (2010). Refugia revisited: individualistic responses of species in space and time. *Proc. R. Soc. B* 277 661–671. 10.1098/rspb.2009.1272 19864280 PMC2842738

[B53] Sun Y. S. Li L. L. Li L. Zou J. B. Liu J. Q. (2015). Distributional dynamics and interspecific gene flow in *Picea likiangensis* and *P. wilsonii* triggered by climate change on the Qinghai-Tibet Plateau. *J. Biogeogr.* 42 475–484. 10.1111/jbi.12434

[B54] Venables W. N. Ripley B. D. (2002). *Modern Applied Statistics with S. Fourth Edition.* New York, NY: Springer 10.1007/978-0-387-21706-2

[B55] Via S. West J. (2008). The genetic mosaic suggests a new role for hitchhiking in ecological speciation. *Mol. Ecol.* 17 4334–4345. 10.1111/j.1365-294X.2008.03921.x 18986504

[B56] Wang L. M. Zhang Y. J. (2011). Discussion on the taxonomic position and nomenclature of Liaodong oak (Fagaceae). *Plant Sci. J.* 29 749–754.

[B57] Wiens J. J. (2004). Speciation and ecology revisited: phylogenetic niche conservatism and the origin of species. *Evolution* 58 193–197. 10.1111/j.0014-3820.2004.tb01586.x 15058732

[B58] Xu X. T. Wang Z. H. Rahbek C. Lessard J. P. Fang J. Y. (2013). Evolutionary history influences the effects of water-energy dynamics on oak diversity in Asia. *J. Biogeogr.* 40 2146–2155. 10.1111/jbi.12149

[B59] Xu X. T. Wang Z. H. Rahbek C. Sanders N. J. Fang J. Y. (2016). Geographical variation in the importance of water and energy for oak diversity. *J. Biogeogr.* 43 279–288. 10.1111/jbi.12620

[B60] Yang J. Di X. Y. Meng X. Feng L. Liu Z. L. Zhao G. F. (2016). Phylogeography and evolution of two closely related oak species (Quercus) from north and northeast China. *Tree Genet. Genomes* 12:89. 10.1007/s11295-016-1044-5 22059561

[B61] Ying J. S. (1994). An analysis of the flora of Qinling Mountain range: its nature, characteristics, and origins. *Acta Phytotaxonomica Sin.* 32 389–410.

[B62] Yu G. Chen X. Ni J. Cheddadi R. Guiot J. Han H. (2000). Palaeovegetation of China a pollen data-based synthesis for the mid-Holocene and last glacial maximum. *J. Biogeogr.* 27 635–664. 10.1046/j.1365-2699.2000.00431.x

[B63] Zeng Y. F. Liao W. J. Petit R. J. Zhang D. Y. (2010). Exploring species limits in two closely related Chinese oaks. *PLoS One* 5:e15529. 10.1371/journal.pone.0015529 21152084 PMC2994836

[B64] Zeng Y. F. Liao W. J. Petit R. J. Zhang D. Y. (2011). Geographic variation in the structure of oak hybrid zones provides insights into the dynamics of speciation. *Mol. Ecol.* 20 4995–5011. 10.1111/j.1365-294X.2011.05354.x 22059561

[B65] Zeng Y. F. Wang W. T. Liao W. J. Wang H. F. Zhang D. Y. (2015). Multiple glacial refugia for cool-temperate deciduous trees in northern East Asia: the Mongolian oak as a case study. *Mol. Ecol.* 24 5676–5691. 10.1111/mec.13408 26439083

[B66] Zhao J. L. Gugger P. F. Xia Y. M. Li Q. J. (2016). Ecological divergence of two closely related *Roscoea* species associated with late Quaternary climate change. *J. Biogeogr.* 43 1990–2001. 10.1111/jbi.12809 21438932

[B67] Zheng B. X. Xu Q. Q. Shen Y. P. (2002). The relationship between climate change and Quaternary glacial cycles on the Qinghai–Tibetan Plateau: review and speculation. *Quat. Int.* 97-98 93–101. 10.1016/S1040-6182(02)00054-X

