# Supplementary figures and images for: Climatic and Soil Factors Shape the Demographical History and Genetic Diversity of a Deciduous Oak (Quercus liaotungensis) in Northern China
Source: Front Plant Sci. 2018 Oct 25;9:1534. doi: 10.3389/fpls.2018.01534 (PMC6209687; doi:10.3389/fpls.2018.01534)

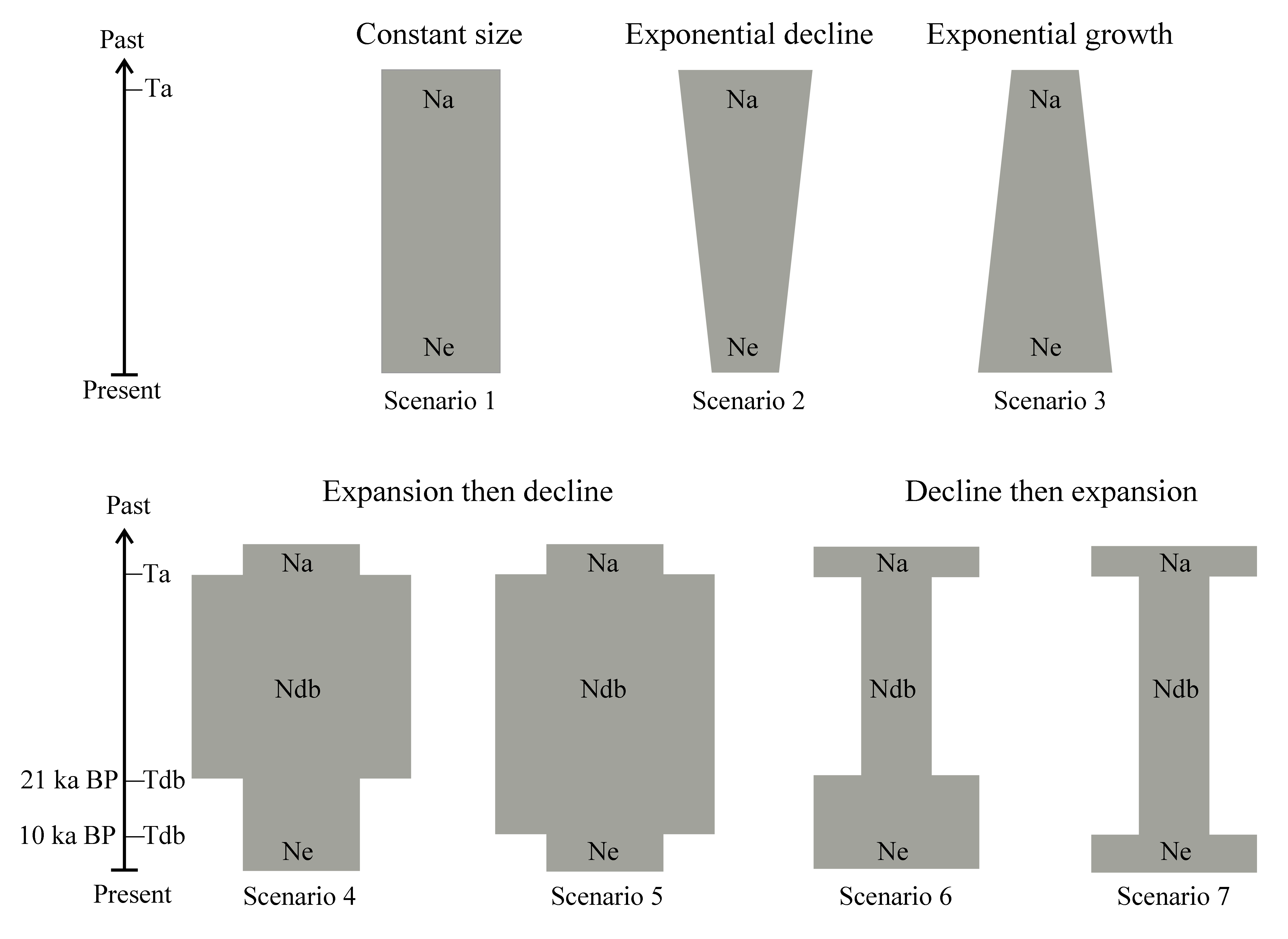

Supplement: FIGURE S1 — Scenarios tested using approximate Bayesian computation (ABC) for global changes in population sizes of Q. liaotungensis. Na, ancestral population size; Ne, current population size; Ndb, population changes between Na and Ne; Tdb, transfer time between Ndb and Ne; Ta, established time of ancestral populations. [file Image_1.TIF]

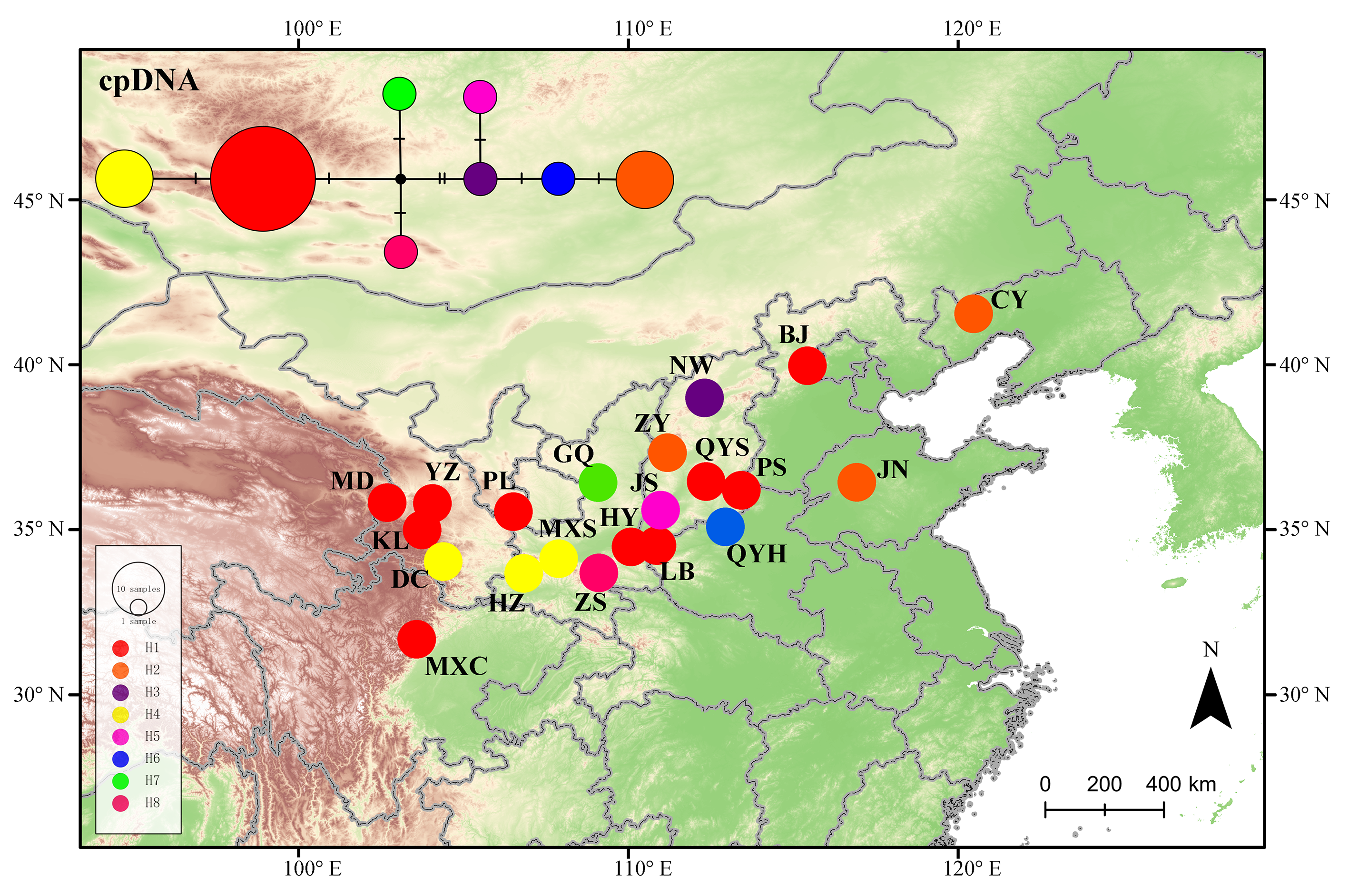

Supplement: FIGURE S2 — Haplotype distributions and network of concatenated chloroplast regions for Quercus liaotungensis in Northern China. Pie charts are proportional to the numbers of samples in each population (five individuals per site), and different pie colors correspond to different haplotypes in the network. [file Image_2.TIF]

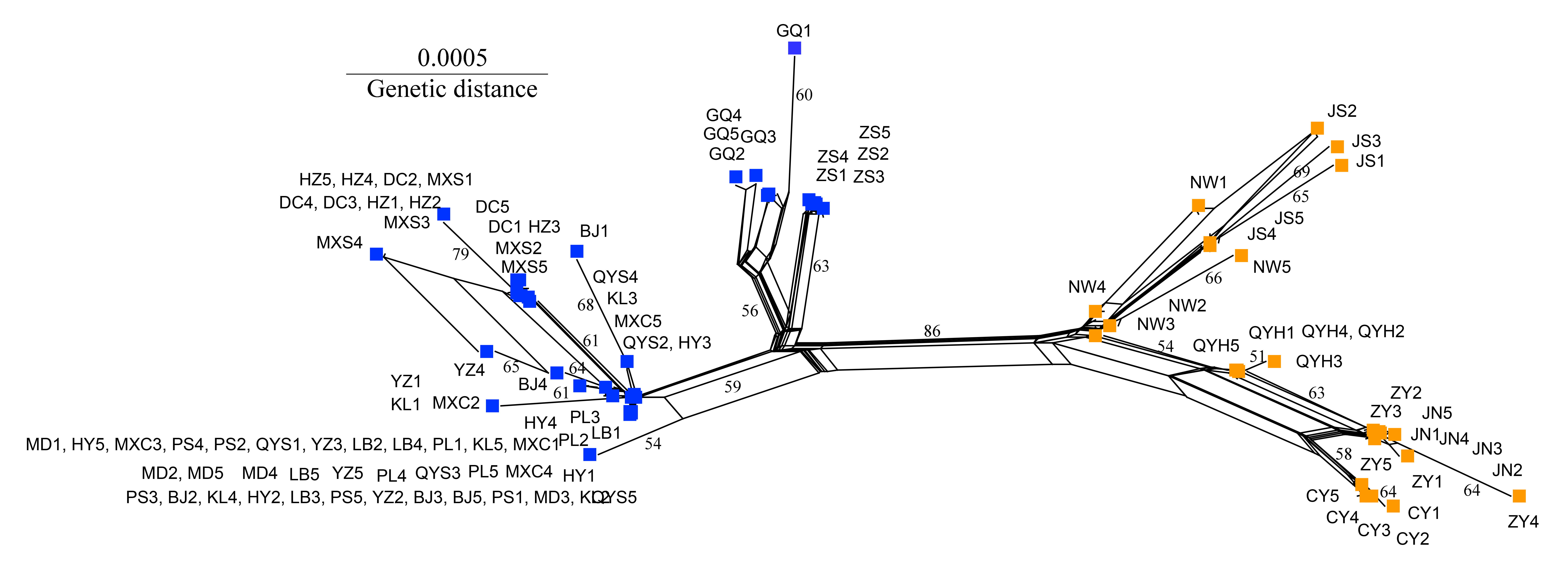

Supplement: FIGURE S3 — Detailed neighbor-net network of all individuals indicates genetic relationships of Quercus liaotungensis. Blue and orange squares represent samples identified in clusters 1 and 2, respectively. Bootstrap values >50 are shown near the branch. [file Image_3.TIF]

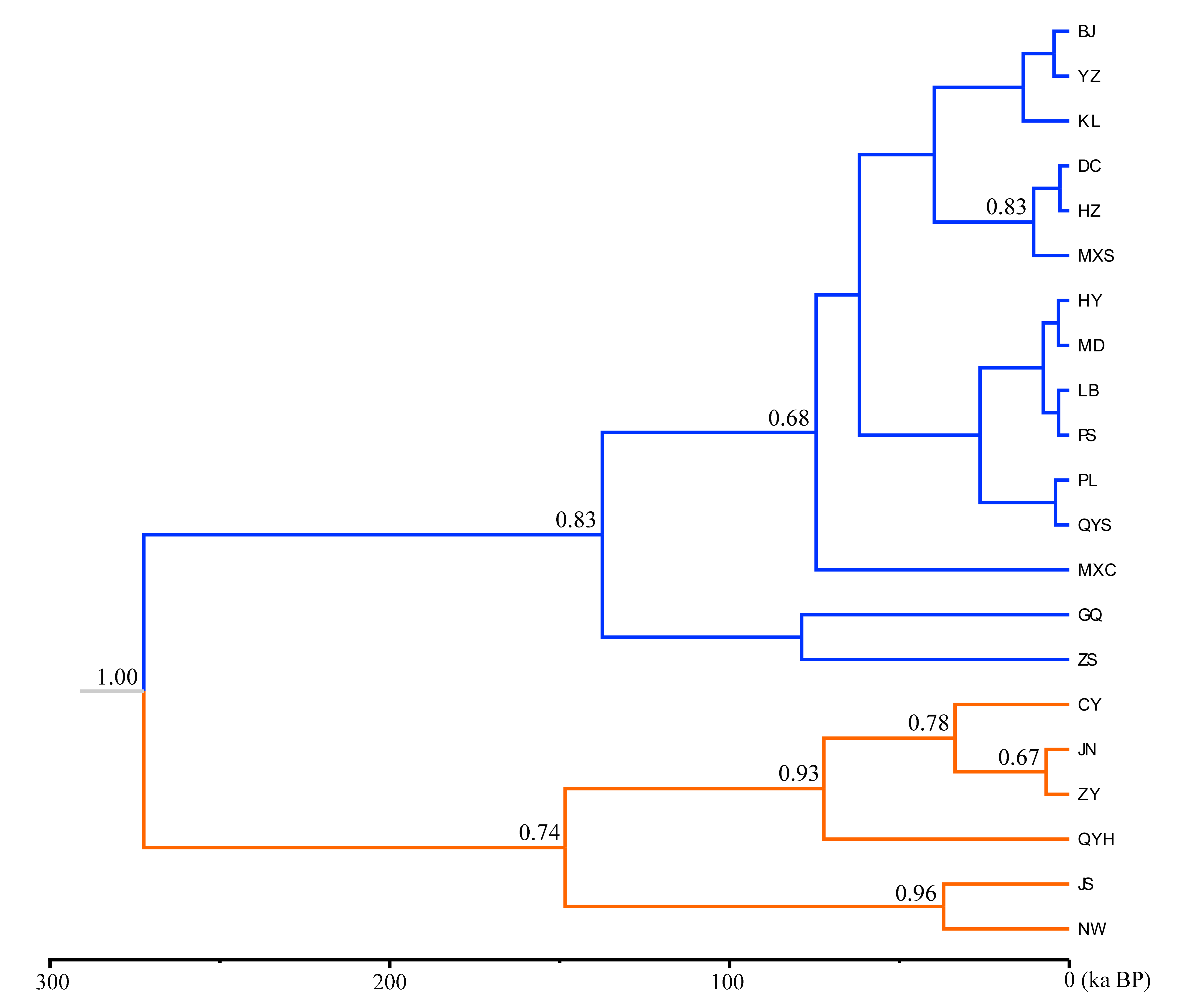

Supplement: FIGURE S4 — Time-estimated phylogenetic tree of 21 populations from Quercus liaotungensis reveals two major clusters using coalescent-based Bayesian analysis. Branches in blue and orange indicate populations belonging to phylogenetic clusters 1 and 2, respectively. Values of posterior probability >0.6 are shown on the branch. [file Image_4.TIF]

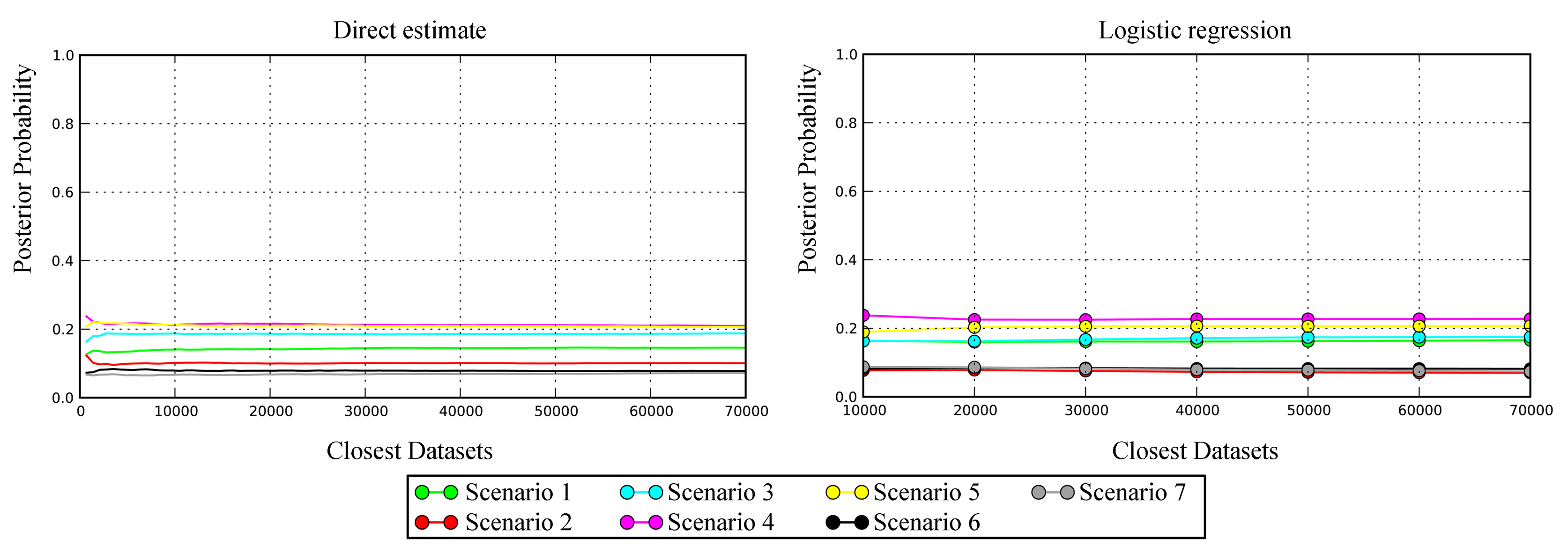

Supplement: FIGURE S5 — Comparison of posterior probabilities of seven simulated scenarios obtained with direct estimate and logistic regression from 1% of closest datasets for approximate Bayesian computation. [file Image_5.TIF]

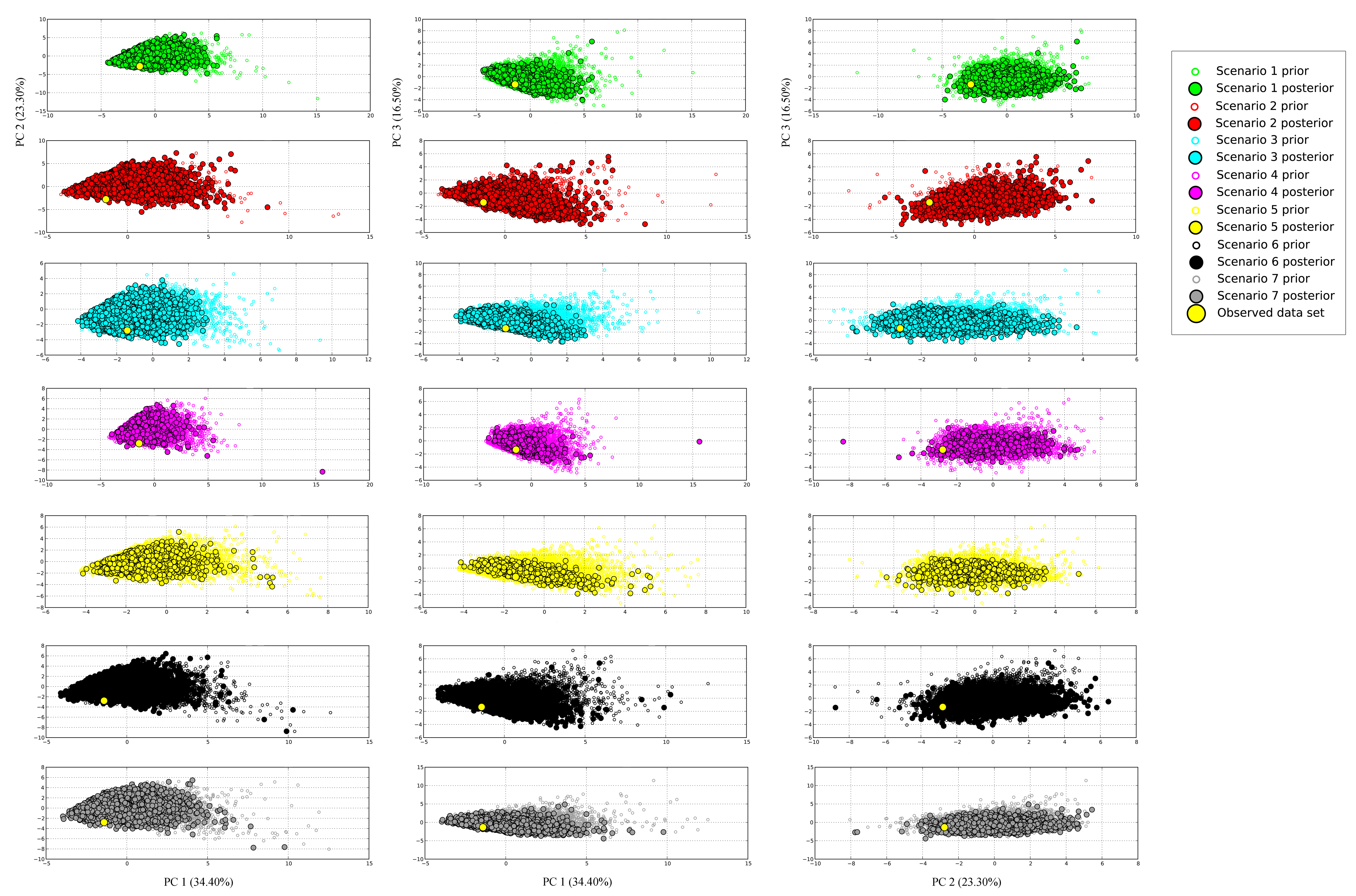

Supplement: FIGURE S6 — Panels of principal component analysis along the first three axes for five summary statistics of seven simulated scenarios for model checking in approximate Bayesian computation. [file Image_6.TIF]
